# Draft genome assemblies for tree pathogens *Phytophthora pseudosyringae* and *Phytophthora boehmeriae*

**DOI:** 10.1093/g3journal/jkab282

**Published:** 2021-08-13

**Authors:** Peter Thorpe, Ramesh R Vetukuri, Pete E Hedley, Jenny Morris, Maximilian A Whisson, Lydia R J Welsh, Stephen C Whisson

**Affiliations:** 1 School of Medicine, University of St Andrews, North Haugh, St Andrews KY16 9TF, UK; 2 Department of Plant Breeding, Swedish University of Agricultural Sciences, Lomma, SE-234 22, Sweden; 3 Cell and Molecular Sciences, James Hutton Institute, Invergowrie, Dundee DD2 5DA, UK; 4 Independent, Dundee DD5 4QT, UK

**Keywords:** tree pathogen, *Phytophthora*, oomycete, effector, RXLR

## Abstract

Species of *Phytophthora*, plant pathogenic eukaryotic microbes, can cause disease on many tree species. Genome sequencing of species from this genus has helped to determine components of their pathogenicity arsenal. Here, we sequenced genomes for two widely distributed species, *Phytophthora pseudosyringae* and* Phytophthora boehmeriae*, yielding genome assemblies of 49 and 40 Mb, respectively. We identified more than 270 candidate disease promoting RXLR effector coding genes for each species, and hundreds of genes encoding candidate plant cell wall degrading carbohydrate active enzymes (CAZymes). These data boost genome sequence representation across the *Phytophthora* genus, and form resources for further study of *Phytophthora* pathogenesis.

## Introduction

*Phytophthora* species are oomycetes, eukaryotic plant pathogens with a filamentous growth habit that superficially resemble fungi. However, they are stramenopiles, phylogenetically separated from the true fungi, belonging to the SAR group (stramenopiles-alveolata-rhizaria) (Cavalier-Smith 2018). Species of *Phytophthora* represent significant threats to food security, causing billions of dollars of losses to key crops such as potato, tomato, and soybean (Tyler 2007; Fry *et al.* 2015). *Phytophthora* species also represent some of the greatest threats to tree and forest health (Hansen *et al.* 2012). Sampling from diseased trees, forest soils, and water sources has led to the identification of many *Phytophthora* species that can infect trees (Jung *et al.* 2017, 2018).

While molecular host-pathogen interactions are intensively researched for a small number of crop pathogenic *Phytophthora* species, such as *Phytophthora* *infestans* (potato late blight) and *Phytophthora* *sojae* (soybean root rot), most tree pathogenic species have not received as much attention (Kamoun *et al.* 2015). Sudden oak death caused by *Phytophthora* *ramorum*, and Jarrah dieback caused by *Phytophthora* *cinnamomi*, are well-known examples of tree pathogenic species with global impact (Grünwald *et al.* 2008; Fisher *et al.* 2012; Hardham and Blackman 2018). Pathogens of woody host plants are present in most, if not all, clades of the *Phytophthora* genus. The genomes of many tree pathogenic species have been sequenced in recent years (for example Feau *et al.* 2016; Studholme *et al.* 2016, 2019; Vetukuri *et al.* 2018), providing resources to predict and compare the pathogenicity factors encoded in these genomes.

Among the most commonly detected *Phytophthora* species in the United Kingdom in diseased plants and soil is *Phytophthora* *pseudosyringae* (Clade 3) (Riddell *et al.* 2019). *P. pseudosyringae* is also present in many other countries (Linzer *et al.* 2009; Scanu *et al.* 2015; Stamler *et al.* 2016; Hansen *et al.* 2017; Khaliq *et al.* 2019). A draft genome sequence was recently reported for *P. pseudosyringae* (McGowan *et al.* 2020), which provided the first view of the pathogenicity arsenal in this species. The only other genome sequence from a Clade 3 species is from *Phytophthora* *pluvialis* (Studholme *et al.* 2016). Another globally distributed species is *Phytophthora* *boehmeriae* (Clade 10) which not only causes disease on trees such as black wattle (*Acacia mearnsii*), but also crops such as cotton and chili (Chowdappa *et al.* 2014; Santos 2016; Wang *et al.* 2020). *Phytophthora* Clade 10 contains few species (Yang *et al.* 2017). While there are multiple genome sequences for different isolates of the tree pathogen *P. kernoviae*, only one other Clade 10 genome is available (Studholme *et al.* 2019).

To build molecular biology resources and enable future gene diversity studies in *P. pseudosyringae*, we generated a further draft genome sequence assembly for this species. To facilitate more robust evolutionary studies into pathogenicity of Clade 10 species in future, we also generated a genome sequence assembly for *P. boehmeriae*.

## Materials and methods

### *Phytophthora* cultures, DNA preparation, and Illumina sequencing

Cultures of *P. pseudosyringae* (SCRP734) and *P. boehmeriae* (SCRP23) from the James Hutton Institute culture collection were maintained on rye agar. Hyphae for both species were grown without shaking in amended lima bean broth (Bruck *et al.* 1981) for 72 h at 20°C, harvested by gravity filtration through 70 µm nylon mesh, and immediately frozen in liquid nitrogen until used for DNA extraction. Genomic DNA from each species was extracted from the frozen hyphae using the protocol of Raeder and Broda (1985), followed by RNaseA treatment and DNeasy (Qiagen) column clean-up using the manufacturer’s protocol. DNA quality was assessed by absorbance at 260 and 280 nm (Nanodrop), and agarose gel electrophoresis. Genomic DNA was fragmented for sequencing library construction by physical shearing using a M220 ultrasonicator (Covaris) as recommended. Libraries for whole-genome sequencing were prepared using the Illumina TruSeq DNA PCR-Free kit, following the manufacturer’s protocol (350 bp insert) and standard indexing. Libraries were sequenced (150 bp, paired-end) on a NextSeq 500 (Illumina) as recommended.

### *Phytophthora* genome assembly and gene prediction

Reads for each species were separately subjected to quality control using trimmomatic (Q15) (Bolger *et al.* 2014). FASTQC (http://www.bioinformatics.babraham.ac.uk/projects/fastqc/) (last accessed 16 August 2021) was used to assess read quality pre and post-read trimming. CLC assembler (version 4.10.86742) (Qiagen CLC Genomics Workbench) was used for a first pass assembly. This assembly was subject to Blobtools (version 1.0) (Laetsch and Blaxter 2017) analysis to identify contaminant contigs. The contigs were DIAMOND-BLASTp (version 0.9.24.125) (Buchfink *et al.* 2015) searched against the NCBI NR database and taxonomically assigned using Blobtools. For final assembly, both genomes were processed through multiple rounds of read filtering/assembly and Blobtools-contamination identification. The final assembly was made with CLC using the contamination filtered reads (as described in Thorpe *et al.* 2018), and was passed as trusted contigs for SPAdes (version 3.13.0) (Bankevich *et al.* 2012). The resulting SPAdes assembly was screened through Blobplots once more. The final contig assembly was then subjected to scaffolding using SSPACE (version 3.0) (Boetzer *et al.* 2011). To reduce false-positive scaffolding, dereplicated reads are needed; the Q15 or greater quality reads already prepared using trimmomatic were dereplicated using PRINSEQ (prinseq-lite-0.20.4) (Schmieder and Edwards 2011). Assemblies were repeat masked using a *de novo* set of *Phytophthora* specific repeats (generated here, as described in Thorpe *et al.* 2018) and repbase (Bao *et al.* 2015). These were modeled from multiple *Phytophthora* genomes. This was then taken further to predict transposons.

Genomes were soft-masked (lower case) using the identified transposon and repetitive regions, see above, using BEDTools (Quinlan and Hall 2010). Funannotate version 1.5.3 (https://github.com/nextgenusfs/funannotate) (last accessed 16 August 2021) was used to predict genes using the genome only mode. Briefly, Funannotate wraps BUSCO2 (Seppey *et al.* 2019) to train Augustus (Hoff and Stanke 2019) which resulted in a gene-level prediction sensitivity and specificity of 0.85 and 0.559, respectively. Genemark-ES (Borodovsky and Lomsadze 2011) was also used to predict genes following DIAMOND-BLAST search against the genome using the Swissprot database (https://www.uniprot.org/statistics/Swiss-Prot) (last accessed 16 August 2021). Moreover, GlimmerHMM (Pertea and Salzberg 2002) and SNAP (Korf 2004) were also used to predict genes. The resulting predictions were then passed into Evidence Modeller (Haas *et al.* 2008). The genes were then functionally annotated (putative assignments) using PFAM (https://pfam.xfam.org/) (last accessed 16 August 2021), InterProScan (https://www.ebi.ac.uk/interpro/search/sequence/) (last accessed 16 August 2021) and Eggnog (Huerta-Cepas *et al.* 2019).

The completeness of each *Phytophthora* genome assembly was assessed by analysis of 234 conserved stramenopile genes, with BUSCO (Seppey *et al.* 2019) run in long mode.

Ploidy level was estimated by mapping the dereplicated Q15 reads to the final genome using BWA-mem (Li 2013) and assessed using nQuire (Weiß *et al.* 2018). nQuire uses several statistical approaches, for example, correlating the SNP distribution with the expected distribution of various known ploidy distributions. The resulting statistical output was then interrogated to determine the most statistically significant ploidy level.

Genome size and heterozygosity were predicted using Genomescope v.2.0 (Ranallo-Benavidez *et al.* 2020) using kmers of *k* = 21. Briefly, using the resulting bam files generated above, kmers were counted using Jellyfish (Marcais and Kingsford 2011), count *m* = 21, and converted into a histogram using Jellyfish histo. The resulting histogram was uploaded to the Genomscope2.0 web interface (http://qb.cshl.edu/genomescope/genomescope2.0/) (last accessed 16 August 2021).

### Secreted proteins, RXLR effectors, and CAZymes

The catalog of secreted proteins (presence of a signal peptide and absence of a transmembrane domain) for each *Phytophthora* genome was predicted by SignalP v4.1 (Nielsen 2017) and Phobius (Käll *et al.* 2004).

To identify genes encoding secreted RXLR effectors, we adopted an inclusive strategy. RXLR effector genes were predicted using three strategies, described in Whisson *et al.* (2007), Win *et al.* (2007), and Bhattacharjee *et al.* (2006). The resulting gene lists were then combined to form a final nonredundant catalog of genes encoding candidate RXLR effectors.

Intergenic distances (bp) were calculated for all genes, and separately for the BUSCO and RXLR effector coding genes. Intergenic distances at 5’ and 3’ ends of genes were then plotted against each other to identify if there was a bias in the intergenic distances for the RXLR effector coding genes.

Candidate secreted crinkle and necrosis (CRN) effectors were predicted using a regular expression search for L[FY]LA[RK] in the predicted proteins from both species sequenced (https://github.com/peterthorpe5/reg_exp_finder/blob/master/crinkler_reg.py) (last accessed 16 August 2021). The positive hits were then aligned using Muscle (Edgar 2004) and inputted to hmmbuild. The resulting Hidden Markov Model was used to interrogate the predicted proteins using hmmsearch (1e^−10^ cut off), to yield the candidate CRNs. A further regular expression search only for the LXLFLAK motif characteristic of CRN effectors (Schornack *et al.* 2010) was also performed (https://github.com/test1932/Regex-script-s-) (last accessed 16 August 2021). Returned sequences were assessed by SignalP v5.0 for the presence of a signal peptide.

Additional regular expression searches were performed for proteins containing the PEP13 elicitor (VWNQPVRGFKVXE) (Brunner *et al.* 2002), the GHRHDWE peptide characteristic of necrosis and ethylene-inducing 1-like (NLP) proteins (Chen *et al.* 2018), and HXGPCEXXXDD found in a class of candidate secreted effector proteins in *P. palmivora* (Evangelisti *et al.* 2017).

Carbohydrate active (CAZy) proteins were predicted through the dbCAN2 web server, using hmmer, DIAMOND-BLAST, and HotPep tools (Zhang *et al.* 2018).

### Alien Index calculation to detect candidate horizontal gene transfer events

Potential horizontal gene transfer (HGT) events in the evolutionary history of the *Phytophthora* species sequenced here were identified through calculation of the Alien Index (AI) for each gene (Gladyshev *et al.* 2008; Flot *et al.* 2013), as described by Thorpe *et al.* (2018). Differences to the published method were that DIAMOND-BLASTP results identified as nonstramenopile were considered as candidate HGT events, and the strict AI threshold value for a gene to be considered as an HGT event was 30.

### BLAST similarity and dN/dS analysis of RXLR effectors

Predicted RXLR effector protein sequences and predicted proteomes were searched against the NCBI NR database. Percentage identity, length of alignment, and bitscore were recorded and binned for each query sequence. Percentage identity, alignment, and bitscore were plotted against number of sequences in each bin.

For comparison with the RXLR effectors from the species sequenced in this report, genome assemblies (including versions) of 27 *Phytophthora* species were used to source RXLR effectors for dN/dS calculations (Supplementary Table S1). As a negative control, the set of genes identified from BUSCO analysis for the species assembled here were analyzed.

Analysis of selection on RXLR and BUSCO proteins was carried out by Orthofinder (Emms and Kelly 2019) and separately a Reciprocal Best BLAST Hit (RBBH) clustering network, as described in Thorpe *et al.* (2016). Briefly, RBBH searches were performed between all species. The results were then clustered using MCL (version 14-137) (Dongen 2000) using inflation value *I* = 6, which resulted in an RBBH clustering network. dN/dS analysis was performed as described in Thorpe *et al.* (2016) with slight modification. Briefly, amino acid sequences for each cluster were aligned using Muscle (Edgar 2004) and badseq_remover.pl (https://github.com/dukecomeback/bad-sequence-remover) (last accessed 16 August 2021) was used to remove sequences too divergent from each other. Then the alignment was refined using Muscle. The aligned amino acid sequence was back translated to its original nucleotide sequence, preserving its alignment information. The nucleotide aligned clusters were then filtered for the most informative coding regions, and indels removed using trimAI (no_gaps) (Capella-Gutiérrez *et al.* 2009). On clusters with three or more members, Codonphyml (Gil *et al.* 2013) was used to perform dN/dS analysis. Detailed method and scripts can be found at the github page listed in the data availability statement.

## Results and discussion

### Genome assembly

Illumina sequencing yielded 118,669,161 and 168,739,903 raw reads for *P. pseudosyringae* and* P. boehmeriae*, respectively. Our 48.9 Mb assembly for *P. pseudosyringae* is broadly similar in size to that reported for a different isolate by McGowan *et al.* (2020), but the N50 value of 76 kb for the assembly reported here is threefold greater (Table 1). The *P. boehmeriae* genome assembly, at 40.0 Mb, is similar in size to other sequenced Clade 10 *Phytophthora* species (Sambles *et al.* 2015; Studholme *et al.* 2019).

**Table 1:** Genome assembly and analysis statistics for *P. pseudosyringae* and* P. boehmeriae*

	*P. pseudosyringae*	*P. boehmeriae*
Culture accession	SCRP734	SCRP23
Host plant	*Fagus sylvatica* (European beech)	*Gossypium hirsutum* (cotton)
Country and year	Italy, 2003	China, 1998
Assembled genome size	48,944,789	39,965,592
Predicted haploid genome size (k-mer)	52,169,164	46,223,661
Estimated coverage	440×	828×
GC content	0.546	0.509
N50	76,110	55,354
Mean Scaffold Size	17,656	13,277
Longest Scaffold	415,940	354,378
Number of scaffolds greater than length 200 bp	2,772	3,010
% Heterozygosity	0.01%	0.03%
Predicted genes	15,624	12,121
Secreted proteins	1,599	1,459
RXLR effectors	279	380
CAZy proteins	565	503

Our estimation of heterozygosity in the isolates of the two species sequenced here showed low levels for *P. boehmeriae* and* P. pseudosyringae* (0.01 and 0.03%, respectively). Both *P. pseudosyringae* and* P. boehmeriae* are homothallic (inbreeding), and the observed low levels of heterozygosity are consistent with this.

Genome sizes estimated from sequence reads were 52.1 and 46.2 Mb for *P. pseudosyringae* and* P. boehmeriae*, respectively. These estimates suggest that our assemblies for *P. pseudosyringae* and* P. boehmeriae* are largely representative of the genome content. Discrepancy in genome size estimates and assembly sizes may be resolved through use of longer read sequencing technologies and flow cytometry (Cui *et al.* 2019; Lee *et al.* 2020; van Poucke *et al.* 2021). Genome sizes of *Phytophthora* species, estimated by flow cytometry, were typically larger than the genome assembly sizes from sequencing (McGowan and Fitzpatrick 2017; van Poucke *et al.* 2021). Flow cytometry estimated a haploid genome size for *P. pseudosyringae* ranging from 72 to 86 Mb (van Poucke *et al.* 2021); no data are available for *P. boehmeriae*.

The repetitive DNA content in *Phytophthora* genomes can be highly variable, ranging from 74% for *P. infestans* (Haas *et al.* 2009) to 15% in *P. plurivora* (Vetukuri *et al.* 2018). These repeats encompass a diverse range of DNA sequences, but are primarily made up of mobile elements, either intact or degraded. We estimated the proportion of mobile element repeats in the two genome assemblies reported here at 22.4% (10.9 Mb) for *P. pseudosyringae* and 13.3% (5.3 Mb) for *P. boehmeriae*.

*Phytophthora* species are at least diploid during asexual stages and may have elevated ploidy. We used nQuire to estimate ploidy from sequence data, where the smallest ΔlogL is accepted as the ploidy level. We found the greatest support for tetraploidy in *P. pseudosyringae* (ΔlogL = 1534) and *P. boehmeriae* (ΔlogL = 603.8).

### Gene prediction, and BUSCO v2 estimation of genome completeness

We predicted 15,624 genes for *P. pseudosyringae* and 12,121 genes for *P. boehmeriae*. For *P. pseudosyringae*, this is a similar gene number to that previously predicted by McGowan *et al.* (2020). The completeness of the genome assemblies was estimated using a set of 234 conserved stramenopile genes (BUSCO v2; Table 2). This showed a high degree of gene representation for *P. pseudosyringae* (92.3%) and *P. boehmeriae* (90.2%). The assemblies for *P. pseudosyringae* and* P. boehmeriae* had no duplicated or fragmented BUSCO genes (Table 2).

**Table 2:** BUSCO analysis of *P. pseudosyringae* and *P. boehmeriae* genome assemblies

	*P. pseudosyringae*	*P. boehmeriae*
Complete	216 (92.3%)	211 (90.2%)
Complete, single copy	216	211
Completed, duplicated	0	0
Fragmented	0	0
Missing	18 (7.7%)	23 (9.8%)

**Figure 1:**
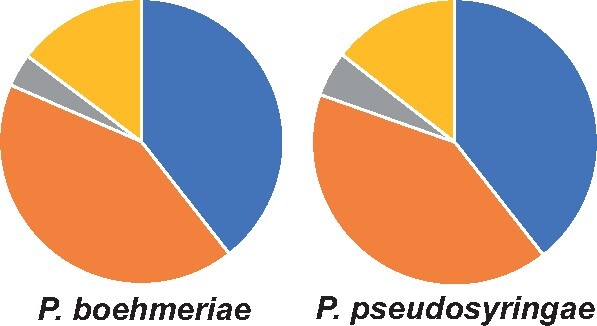
Classes of gene duplications in *P. boehmeriae* and *P. pseudosyringae*. Single copy genes are shown in blue, dispersed copies in orange, proximal duplications in grey, and tandem duplications in yellow. Segmental duplications represent 0.1% or less of gene duplications and are not shown here. Genomes of *P. boehmeriae* and *P. pseudosyringae* have similar proportions of single copy and different classes of duplications.

Gene duplication was also assessed for both species, identifying single copy, dispersed copies, proximal duplications, tandem duplications, and segmental duplications. *P. boehmeriae* and* P. pseudosyringae* have similar proportions of single-copy and duplicated genes (Figure 1; Supplementary Table S2).

### Pathogenicity genes

*Phytophthora* species are known to deploy a diverse array of secreted proteins to facilitate infection (Schornack *et al.* 2009; McGowan and Fitzpatrick 2017). We predicted signal peptides for a total of 1,599 and 1,459 proteins from *P. pseudosyringae* and* P. boehmeriae*, respectively.

A major component of the pathogenicity arsenal of *Phytophthora* species are a large group of secreted proteins that are translocated into plant cells to exert their function. They are characterized by a conserved RXLR peptide motif (arginine-any amino acid-leucine-arginine) within the N-terminal 50 amino acids (Whisson *et al.* 2007; Wang *et al.* 2017). We adopted an inclusive strategy for identifying candidate RXLR effector coding genes, incorporating results from three different search strategies. We identified 279 and 380 candidate RXLR effectors from *P. pseudosyringae* and* P. boehmeriae*, respectively (Supplementary Table S3). The candidate RXLR effector number for *P. pseudosyringae* is higher than that determined by McGowan *et al.* (2020) due to the more inclusive prediction strategy used here. The list of RXLR effector candidates will likely include some false positive predictions, but these can be eliminated using future transcriptome experiments of plant infection; RXLR effectors are typically up-regulated during infection (Haas *et al.* 2009; Jupe *et al.* 2013). From our RXLR predictions, we identified 118 and 276 proteins possessing both complete RXLR and downstream EER motifs from *P. pseudosyringae* and* P. boehmeriae*, respectively. Both of these motifs have been demonstrated to have a role in translocating effectors into plant cells (Whisson *et al.* 2007; Dou *et al.* 2008), and the RXLR motif represents a protease cleavage site (Wawra *et al.* 2017).

**Figure 2:**
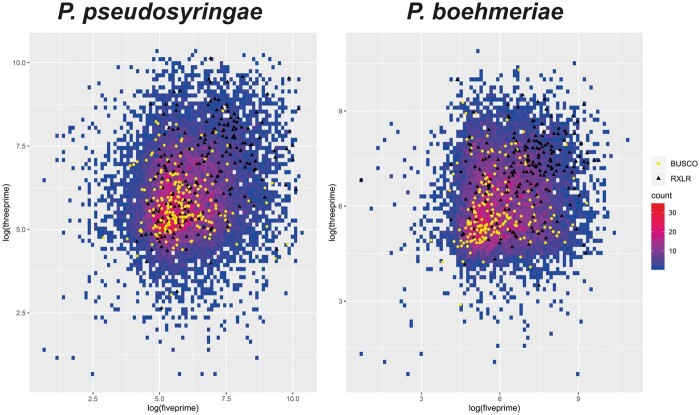
Plots of 5’ against 3’ intergenic distances (log_10_) for genes from *P. pseudosyringae* and *P. boehmeriae*. Richness of gene density for intergenic distances is represented by color scale ranging from blue (low) to red (high). Genes encoding RXLR effector proteins are shown as black triangles; BUSCO genes are shown as yellow dots. Only genes for which both 5’ and 3’ intergenic distances could be calculated are shown.

In other *Phytophthora* genomes, RXLR effectors predominantly reside in gene poor, repeat rich regions, which is reflected in larger intergenic distances (Haas *et al.* 2009; Vetukuri *et al.* 2018). We also determined the 5’ and 3’ intergenic distances from RXLR effector coding genes from *P. pseudosyringae* and* P. boehmeriae* and plotted them against all genes from the genomes, and against the set of genes identified in BUSCO analysis. Only genes for which both 5’ and 3’ intergenic distances could be calculated were included in plots. This revealed that, similar to other examined *Phytophthora* species, the intergenic distances for RXLR effector coding genes in these species are greater than those for the core set of genes (Figure 2; Supplementary Table S4).

Using regular expression and HMM searches to predict candidate secreted CRN effectors, we predicted only a single candidate in *P. pseudosyringae*, and two in *P. boehmeriae* (Supplementary Table S5). This was surprising, as most *Phytophthora* genomes sequenced to date encode numerous candidate CRN effectors (McGowan and Fitzpatrick 2017). Many predicted CRN-family proteins do not possess predicted signal peptides (McGowan and Fitzpatrick 2017) and here we found that none of the three CRN candidates possessed a predicted signal peptide. McGowan *et al.* (2020) manually curated 90 CRN candidates in *P. pseudosyringae*, but only 37 were considered to be secreted effector proteins. Our findings suggest that CRN proteins are not major contributors to pathogenicity in the two species sequenced.

*Phytophthora* species are also known to possess multiple genes encoding proteins that can cause plant cell death, either by cytotoxicity or through elicitation of plant immune responses. Regular expression searches for two families of these proteins revealed four candidate PEP13 elicitor containing proteins in *P. pseudosyringae* and* P. boehmeriae* (Supplementary Table S5). For cytotoxic NLP1-like proteins, seven candidates were identified in *P. pseudosyringae* (six predicted secreted), and 14 in *P. boehmeriae* (12 secreted) (Supplementary Table S5).

Evangelisti *et al.* (2017) identified a class of 42 predicted secreted proteins from *P. palmivora* that contained a conserved HXGPCEXXXDD peptide motif. Searches of the predicted proteomes of the two species sequenced here identified 34 proteins containing this motif from *P. pseudosyringae* (29 predicted secreted), and 42 from *P. boehmeriae* (36 secreted) (Supplementary Table S5). That the motif is conserved in similar numbers of proteins in these species from different clades of the genus, and a high proportion are predicted to be secreted, suggests that they may be a class of candidate effector proteins important for *Phytophthora* pathogenicity.

Another significant set of genes involved in pathogenicity encode carbohydrate active (CAZy) proteins, especially those with potential enzymatic activity for digesting plant cell walls, allowing pathogen ingress. *Phytophthora* genomes are predicted to encode hundreds of CAZymes, often present as gene families of closely related members (Ospina-Giraldo *et al.* 2010; Brouwer *et al.* 2014; McGowan and Fitzpatrick 2017). We used three CAZy prediction tools within dbCAN2 to identify candidate CAZy proteins and included all positive returned sequences as candidates, since *Phytophthora* proteins may be more divergent than many CAZy proteins represented in databases. We predicted 565 and 503 CAZy proteins for *P. pseudosyringae* and* P. boehmeriae*, respectively. Of particular interest are the lytic polysaccharide monoxygenases (within the auxiliary activity grouping), glycoside hydrolases, polysaccharide lyases, and carbohydrate esterases (Supplementary Table S6). The number of proteins predicted for each CAZy family was broadly similar in the two *Phytophthora* species sequenced, but with some differences that may reflect the pathogenic niche for each species. For example, *P. pseudosyringae* possesses single secreted lytic cellulose monooxygenase (AA16) and secreted cellulose binding (CBM1) proteins, while *P. boehmeriae* possesses four of each. Similarly, *P. boehmeriae* possesses a single secreted glycoside hydrolase family 10 protein, while *P. pseudosyringae* possesses four.

### Evidence for HGT

During their evolution, the oomycetes may have acquired genes from other kingdoms, with genes potentially transferred horizontally from fungi and bacteria to oomycetes (Morris *et al.* 2009; Richards *et al.* 2011; McCarthy and Fitzpatrick 2016). We carried out a strict analysis of potential HGT events in the two genomes presented here, using a high AI threshold value (Supplementary Table S7). Our approach identified four potential HGT events in *P. pseudosyringae*, all but one from eukaryotic sources. These encompassed proteins with similarity to a TPR/SEL1 repeat protein (PHYPSEUDO_011742), a glycoside hydrolase family 63 (PHYPSEUDO_013022), an alpha/beta hydrolase (PHYPSEUDO_013053), and an M54 peptidase (PHYPSEUDO_014249). A single virus (Catovirus) to oomycete HGT event was identified for *P. boehmeriae* (mRNA capping enzyme; PHYBOEH_005573). Whether these candidate HGTs contribute significantly to pathogenicity remains to be determined experimentally.

### Analysis of selection on RXLR effectors and BUSCOs

**Figure 3:**
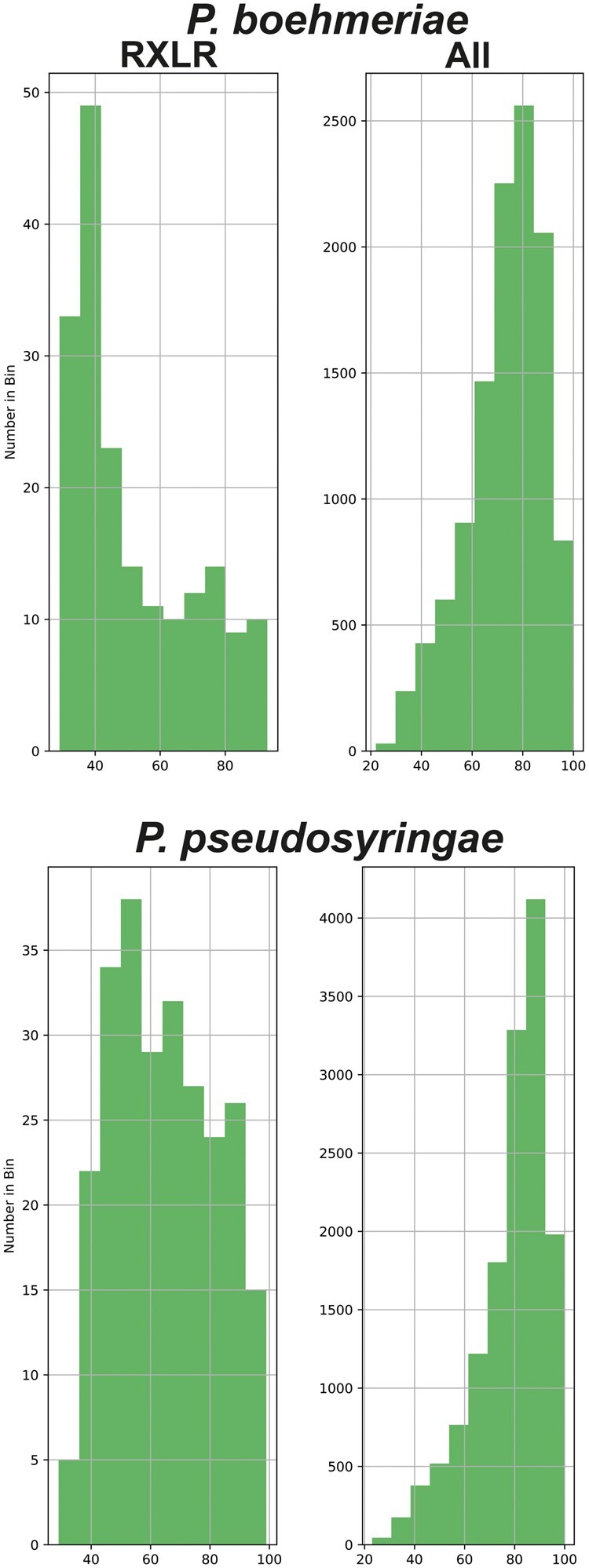
Percentage identity of *P. boehmeriae* (top) and *P. pseudosyringae* (bottom) proteins when BLASTP searched against GenBank non-redundant sequences. Percentage identity (*x*-axis) is plotted against number in each bin (*y*-axis) for RXLR effector proteins, and entire predicted proteome.

The RXLR effectors encoded by *Phytophthora* genomes are considered to be evolving at a greater rate than core ortholog proteins (Win *et al.* 2007). This may be reflected in characteristics such as lower levels of sequence identity with orthologous proteins and shorter length of sequence similarity. When the RXLR effector complements and total predicted proteomes from both genomes in this report were searched against the NCBI NR database, the RXLR effectors exhibited lower levels of sequence identity, lower alignment lengths, and lower bitscores (Figure 3; Supplementary Figure S1). The total predicted proteome showed the opposite trend to the RXLR effectors, with higher levels of sequence identity (Figure 3; Supplementary Figure S1).

The more rapid rate of evolution of RXLR effectors has been reflected in a dN/dS score greater than 1.0 (Win *et al.* 2007). We evaluated dN/dS scores for the two genome assemblies reported here, using both Orthofinder and a reciprocal best blast hit (RBBH) clustering network, the latter being a stricter method. For comparison, we also performed this analysis on the BUSCO orthologs identified when analyzing gene representation in the two genomes. Using Orthofinder, only one gene in the BUSCO gene set had a dN/dS value greater than 1, signifying positive selection (PHYPSEUDO_008773; PITH domain protein; dN/dS = 1.6). Using RBBH, no genes in the BUSCO set showed evidence for positive selection. Using Orthofinder and all RXLR coding genes from both genomes, we found 238 RXLR coding genes were present in clusters with at least two other genes, of which 54 genes showed evidence of positive selection (dN/dS > 1.0) (Supplementary Table S8). RXLR effectors from *P. pseudosyringae* were most highly represented (44) among those exhibiting positive selection, with over four-fold fewer from *P. boehmeriae* (10). The RBBH strategy revealed only three RXLR genes with evidence for positive selection, two from *P. pseudosyringae* (PHYPSEUDO_006359 and PHYPSEUDO_007604) and one from *P. boehmeriae* (PHYBOEH_007729). In other *Phytophthora* species, the number of candidate RXLR effectors exhibiting a signature of positive selection has ranged from as few as one in *P. plurivora* (Vetukuri *et al.* 2018) to greater than 20 in *P. ramorum* (Win *et al.* 2007). The effectors with the greatest level of positive selection are candidates for further studies into their function in disease development.

## Conclusions

Genomes from two *Phytophthora* species that can infect trees have been sequenced and assembled. Our assembly for *P. pseudosyringae* is the most complete. Less fragmented genome assemblies may be achieved in the future by using longer read sequencing. The *P. pseudosyringae* strain used here is the second for this species to be sequenced and will begin to provide insights into gene diversity in this globally prevalent *Phytophthora* species. The genome sequence for *P. boehmeriae* will add to the genome sequencing coverage in Clade 10, which includes the important tree pathogen *P. kernoviae*, and provide a resource for evolutionary studies within this clade and the genus. The candidate pathogenicity proteins identified here will provide a basis for further experimental research, such as transcriptomic analyses and effector function assays, to gain a deeper understanding of *Phytophthora* pathogenesis on trees.

## Data availability

Data in this publication have been deposited at NCBI GenBank: *P. pseudosyringae* (BioProject PRJNA702035; raw data SRX10106440; assembly JAGDFM000000000), and *P. boehmeriae* (BioProject PRJNA702033; raw data SRX10106096; assembly JAGDFL000000000). Assembled genomes, predicted transcriptomes and proteomes, and annotations are also publicly available at https://doi.org/10.5281/zenodo.4554917 (last accessed 16 August 2021). Scripts used to analyze the data are publicly available at https://github.com/peterthorpe5/genomes_tree_phyto_pathogens (last accessed 16 August 2021) and https://github.com/test1932/Regex-script-s- (last accessed 16 August 2021).

Supplementary material is available at *G3 online*.

## Supplementary Material

jkab282_Supplementary_Data

## References

[jkab282-B1] Bankevich A , Nurk S, Antipov D, Gurevich AA, Dvorkin M, et al 2012. SPAdes: a new genome assembly algorithm and its applications to single-cell sequencing. J Comput Biol. 19:455–477. 22506599 10.1089/cmb.2012.0021 PMC3342519

[jkab282-B2] Bao W , Kojima KK, Kohany O. 2015. Repbase update, a database of repetitive elements in eukaryotic genomes. Mob DNA. 6:11. 26045719 10.1186/s13100-015-0041-9 PMC4455052

[jkab282-B3] Bhattacharjee S , Hiller NL, Liolios K, Win J, Kanneganti TD, et al 2006. The malarial host-targeting signal is conserved in the Irish potato famine pathogen. PLoS Pathog. 2:e50. 16733545 10.1371/journal.ppat.0020050 PMC1464399

[jkab282-B4] Boetzer M , Henkel CV, Jansen HJ, Butler D, Pirovano W. 2011. Scaffolding pre-assembled contigs using SSPACE. Bioinformatics. 27:578–579. 21149342 10.1093/bioinformatics/btq683

[jkab282-B5] Bolger AM , Lohse M, Usadel B. 2014. Trimmomatic: a flexible trimmer for Illumina sequence data. Bioinformatics. 30:2114–2120. 24695404 10.1093/bioinformatics/btu170 PMC4103590

[jkab282-B6] Borodovsky M , Lomsadze A. 2011. Eukaryotic gene prediction using GeneMark.hmm-E and GeneMark-ES. Curr Protoc Bioinformatics. 35:4.6.1-4.6.10. 10.1002/0471250953.bi0406s35 PMC3204378 21901742

[jkab282-B7] Brouwer H , Coutinho PM, Henrissat B, de Vries RP. 2014. Carbohydrate-related enzymes of important Phytophthora plant pathogens. Fungal Genet Biol. 72:192–200. 25192612 10.1016/j.fgb.2014.08.011

[jkab282-B8] Bruck RI , Fry WE, Apple AE, Mundt CC. 1981. Effect of protectant fungicides on the developmental stages of *Phytophthora infestans* in potato foliage. Phytopathology. 71:164–166.

[jkab282-B9] Brunner F , Rosahl S, Lee J, Rudd JJ, Geiler C, et al 2002. Pep-13, a plant defense-inducing pathogen-associated pattern from Phytophthora transglutaminases. EMBO J. 21:6681–6688. 12485989 10.1093/emboj/cdf667 PMC139088

[jkab282-B10] Buchfink B , Xie C, Huson DH. 2015. Fast and sensitive protein alignment using DIAMOND. Nat Methods. 12:59–60. 25402007 10.1038/nmeth.3176

[jkab282-B11] Capella-Gutiérrez S , Silla-Martínez JM, Gabaldón T. 2009. trimAl: a tool for automated alignment trimming in large-scale phylogenetic analyses. Bioinformatics. 25:1972–1973. 19505945 10.1093/bioinformatics/btp348 PMC2712344

[jkab282-B12] Cavalier-Smith T. 2018. Kingdom chromista and its eight phyla: a new synthesis emphasising periplastid protein targeting, cytoskeletal and periplastid evolution, and ancient divergences. Protoplasma. 255:297–357. 28875267 10.1007/s00709-017-1147-3 PMC5756292

[jkab282-B13] Chen XR , Huang SX, Zhang Y, Sheng GL, Li YP, et al 2018. Identification and functional analysis of the NLP-encoding genes from the phytopathogenic oomycete *Phytophthora capsici*. Mol Genet Genomics. 293:931–943. 29572661 10.1007/s00438-018-1432-7

[jkab282-B14] Chowdappa P , Madhura S, Kumar BJN, Kumar SPM, Hema KR. 2014. *Phytophthora boehmeriae* revealed as the dominant pathogen responsible for severe foliar blight of *Capsicum annuum* in south India. Plant Dis. 98:90–98. 30708597 10.1094/PDIS-06-13-0601-RE

[jkab282-B15] Cui C , Herlihy JH, Bombarely A, McDowell JM, Haak DC. 2019. Draft assembly of *Phytophthora capsici* from long-read sequencing uncovers complexity. Mol Plant Microbe Interact. 32:1559–1563. 31479390 10.1094/MPMI-04-19-0103-TA

[jkab282-B16] Dongen S. 2000. Graph Clustering by Flow Simulation [PhD thesis]. The Netherlands: University of Utrecht.

[jkab282-B17] Dou D , Kale SD, Wang X, Jiang RH, Bruce NA, et al 2008. RXLR-mediated entry of *Phytophthora sojae* effector Avr1b into soybean cells does not require pathogen-encoded machinery. Plant Cell. 20:1930–1947. 18621946 10.1105/tpc.107.056093 PMC2518231

[jkab282-B18] Edgar RC. 2004. MUSCLE: a multiple sequence alignment method with reduced time and space complexity. BMC Bioinformatics. 5:113. 15318951 10.1186/1471-2105-5-113 PMC517706

[jkab282-B19] Emms DM , Kelly S. 2019. OrthoFinder: phylogenetic orthology inference for comparative genomics. Genome Biol. 20:238–214. 31727128 10.1186/s13059-019-1832-y PMC6857279

[jkab282-B20] Evangelisti E , Gogleva A, Hainaux T, Doumane M, Tulin F, et al 2017. Time-resolved dual transcriptomics reveal early induced *Nicotiana benthamiana* root genes and conserved infection-promoting *Phytophthora palmivora* effectors. BMC Biol. 15:39. 28494759 10.1186/s12915-017-0379-1 PMC5427549

[jkab282-B21] Feau N , Taylor G, Dale AL, Dhillon B, Bilodeau GJ, et al 2016. Genome sequences of six Phytophthora species threatening forest ecosystems. Genom Data. 10:85–88. 27752469 10.1016/j.gdata.2016.09.013 PMC5061060

[jkab282-B22] Fisher MC , Henk DA, Briggs CJ, Brownstein JS, Madoff LC, et al 2012. Emerging fungal threats to animal, plant and ecosystem health. Nature. 484:186–194. 22498624 10.1038/nature10947 PMC3821985

[jkab282-B23] Flot JF , Hespeels B, Li X, Noel B, Arkhipova I, et al 2013. Genomic evidence for ameiotic evolution in the bdelloid rotifer Adineta vaga. Nature. 500:453–457. 23873043 10.1038/nature12326

[jkab282-B24] Fry WE , Birch PR, Judelson HS, Grünwald NJ, Danies G, et al 2015. Five reasons to consider *Phytophthora infestans* a reemerging pathogen. Phytopathology. 105:966–981. 25760519 10.1094/PHYTO-01-15-0005-FI

[jkab282-B25] Gil M , Zanetti MS, Zoller S, Anisimova M. 2013. CodonPhyML: fast maximum likelihood phylogeny estimation under codon substitution models. Mol Biol Evol. 30:1270–1280. 23436912 10.1093/molbev/mst034 PMC3649670

[jkab282-B26] Gladyshev EA , Meselson M, Arkhipova IR. 2008. Massive horizontal gene transfer in bdelloid rotifers. Science. 320:1210–1213. 18511688 10.1126/science.1156407

[jkab282-B27] Grünwald NJ , Goss EM, Press CM. 2008. *Phytophthora ramorum*: a pathogen with a remarkably wide host range causing sudden oak death on oaks and ramorum blight on woody ornamentals. Mol Plant Pathol. 9:729–740. 19019002 10.1111/j.1364-3703.2008.00500.x PMC6640315

[jkab282-B28] Haas BJ , Kamoun S, Zody MC, Jiang RH, Handsaker RE, et al 2009. Genome sequence and analysis of the Irish potato famine pathogen *Phytophthora infestans*. Nature. 461:393–398. 19741609 10.1038/nature08358

[jkab282-B29] Haas BJ , Salzberg SL, Zhu W, Pertea M, Allen JE, et al 2008. Automated eukaryotic gene structure annotation using evidence modeler and the program to assemble spliced alignments. Genome Biol. 9:R7. 18190707 10.1186/gb-2008-9-1-r7 PMC2395244

[jkab282-B30] Hansen EM , Reeser PW, Sutton W. 2012. Phytophthora beyond agriculture. Annu Rev Phytopathol. 50:359–378. 22681450 10.1146/annurev-phyto-081211-172946

[jkab282-B31] Hansen EM , Reeser PW, Sutton W. 2017. Ecology and pathology of Phytophthora ITS clade 3 species in forests in western Oregon, USA. Mycologia. 109:100–114. 28402782 10.1080/00275514.2016.1273622

[jkab282-B32] Hardham AR , Blackman LM. 2018. Phytophthora cinnamomi. Mol Plant Pathol. 19:260–285. 28519717 10.1111/mpp.12568 PMC6637996

[jkab282-B33] Hoff KJ , Stanke M. 2019. Predicting genes in single genomes with AUGUSTUS. Curr Protoc Bioinformatics. 65:e57. 30466165 10.1002/cpbi.57

[jkab282-B34] Huerta-Cepas J , Szklarczyk D, Heller D, Hernández-Plaza A, Forslund SK, et al 2019. eggNOG 5.0: a hierarchical, functionally and phylogenetically annotated orthology resource based on 5090 organisms and 2502 viruses. Nucleic Acids Res. 47:D309–D314. 30418610 10.1093/nar/gky1085 PMC6324079

[jkab282-B35] Jung T , Jung MH, Cacciola SO, Cech T, Bakonyi J, et al 2017. Multiple new cryptic pathogenic Phytophthora species from Fagaceae forests in Austria, Italy and Portugal. IMA Fungus. 8:219–244. 29242773 10.5598/imafungus.2017.08.02.02 PMC5729710

[jkab282-B36] Jung T , Pérez-Sierra A, Durán A, Jung MH, Balci B, et al 2018. Canker and decline diseases caused by soil- and airborne Phytophthora species in forests and woodlands. Persoonia. 40:182–220. 30505001 10.3767/persoonia.2018.40.08 PMC6146643

[jkab282-B37] Jupe J , Stam R, Howden AJ, Morris JA, Zhang R, et al 2013. *Phytophthora capsici*-tomato interaction features dramatic shifts in gene expression associated with a hemi-biotrophic lifestyle. Genome Biol. 14:R63. 23799990 10.1186/gb-2013-14-6-r63 PMC4054836

[jkab282-B38] Käll L , Krogh A, Sonnhammer EL. 2004. A combined transmembrane topology and signal peptide prediction method. J Mol Biol. 338:1027–1036. 15111065 10.1016/j.jmb.2004.03.016

[jkab282-B39] Kamoun S , Furzer O, Jones JD, Judelson HS, Ali GS, et al 2015. The top 10 oomycete pathogens in molecular plant pathology. Mol Plant Pathol. 16:413–434. 25178392 10.1111/mpp.12190 PMC6638381

[jkab282-B40] Khaliq I , St G, Hardy EJ, McDougall KL, Burgess TI. 2019. Phytophthora species isolated from alpine and sub-alpine regions of Australia, including the description of two new species; *Phytophthora cacuminis* sp. nov and *Phytophthora oreophila* sp. nov. Fungal Biol. 123:29–41. 30654955 10.1016/j.funbio.2018.10.006

[jkab282-B41] Korf I. 2004. Gene finding in novel genomes. BMC Bioinformatics. 5:59. 15144565 10.1186/1471-2105-5-59 PMC421630

[jkab282-B42] Laetsch DR , Blaxter ML. 2017. BlobTools: interrogation of genome assemblies. F1000Res. 6:1287.

[jkab282-B43] Lee Y , Cho KS, Seo JH, Sohn SH, Prokchorchik M. 2020. Improved genome sequence and gene annotation resource for the potato late blight pathogen *Phytophthora infestans*. Mol Plant Microbe Interact. 33:1025–1028. 32310703 10.1094/MPMI-02-20-0023-A

[jkab282-B44] Li H. 2013. Aligning sequence reads, clone sequences and assembly contigs with BWA-MEM. arXiv:1303.3997v2 [q-bio.GN].

[jkab282-B45] Linzer RE , Rizzo DM, Cacciola SO, Garbelotto M. 2009. AFLPs detect low genetic diversity for *Phytophthora nemorosa* and *P. pseudosyringae* in the US and Europe. Mycol Res. 113:298–307. 19061958 10.1016/j.mycres.2008.11.004

[jkab282-B46] Marcais G , Kingsford C. 2011. A fast, lock-free approach for efficient parallel counting of occurrences of k-mers. Bioinformatics. 27:764–770. 21217122 10.1093/bioinformatics/btr011 PMC3051319

[jkab282-B47] McCarthy CG , Fitzpatrick DA. 2016. Systematic search for evidence of interdomain horizontal gene transfer from prokaryotes to Oomycete lineages. mSphere. 1:e00195–16. 27642638 10.1128/mSphere.00195-16 PMC5023847

[jkab282-B48] McGowan J , Fitzpatrick DA. 2017. Genomic, network, and phylogenetic analysis of the oomycete effector arsenal. mSphere. 2:e00408–17. 29202039 10.1128/mSphere.00408-17 PMC5700374

[jkab282-B49] McGowan J , O'Hanlon R, Owens RA, Fitzpatrick DA. 2020. Comparative genomic and proteomic analyses of three widespread Phytophthora species: *Phytophthora chlamydospora*, *Phytophthora gonapodyides* and *Phytophthora pseudosyringae*. Microorganisms. 8:E653. 32365808 10.3390/microorganisms8050653 PMC7285336

[jkab282-B50] Morris PF , Schlosser LR, Onasch KD, Wittenschlaeger T, Austin R, et al 2009. Multiple horizontal gene transfer events and domain fusions have created novel regulatory and metabolic networks in the oomycete genome. PLoS One. 4:e6133. 19582169 10.1371/journal.pone.0006133 PMC2705460

[jkab282-B51] Nielsen H. 2017. Predicting secretory proteins with SignalP. Methods Mol Biol. 1611:59-73.) 10.1007/978-1-4939-7015-5_6 28451972

[jkab282-B52] Pertea M , Salzberg SL. 2002. Computational gene finding in plants. Plant Mol Biol. 48:39–48. 11860211

[jkab282-B53] Ospina-Giraldo MD , Griffith JG, Laird EW, Mingora C. 2010. The CAZyome of Phytophthora spp.: a comprehensive analysis of the gene complement coding for carbohydrate-active enzymes in species of the genus Phytophthora. BMC Genomics. 11:525. 20920201 10.1186/1471-2164-11-525 PMC2997016

[jkab282-B54] Quinlan AR , Hall IM. 2010. BEDTools: a flexible suite of utilities for comparing genomic features. Bioinformatics. 26:841–842. 20110278 10.1093/bioinformatics/btq033 PMC2832824

[jkab282-B55] Raeder U , Broda P. 1985. Rapid preparation of DNA from filamentous fungi. Lett Appl Microbiol. 1:17–20.

[jkab282-B56] Ranallo-Benavidez TR , Jaron KS, Schatz MC. 2020. GenomeScope 2.0 and Smudgeplot for reference-free profiling of polyploid genomes. Nat Comm. 11:1432. 10.1038/s41467-020-14998-3 PMC7080791 32188846

[jkab282-B57] Richards TA , Soanes DM, Jones MD, Vasieva O, Leonard G, et al 2011. Horizontal gene transfer facilitated the evolution of plant parasitic mechanisms in the oomycetes. Proc Natl Acad Sci USA. 108:15258–15263. 21878562 10.1073/pnas.1105100108 PMC3174590

[jkab282-B58] Riddell CE , Frederickson-Matika D, Armstrong AC, Elliot M, Forster J, et al 2019. Metabarcoding reveals a high diversity of woody host-associated Phytophthora spp. in soils at public gardens and amenity woodlands in Britain. PeerJ. 7:e6931. 31143546 10.7717/peerj.6931 PMC6526010

[jkab282-B59] Sambles C , Schlenzig A, O'Neill P, Grant M, Studholme DJ. 2015. Draft genome sequences of *Phytophthora kernoviae* and *Phytophthora ramorum* lineage EU2 from Scotland. Genom Data. 6:193–194. 26697371 10.1016/j.gdata.2015.09.010 PMC4664741

[jkab282-B60] Santos AF. 2016. Phytophthora boehmeriae. Forest Phytophthoras. 6:1–4.

[jkab282-B62] Scanu B , Linaldeddu BT, Deidda A, Jung T. 2015. Diversity of Phytophthora species from declining Mediterranean Maquis vegetation, including two new species, *Phytophthora crassamura* and *P. ornamentata* sp. nov. PLoS One. 10:e0143234. 26649428 10.1371/journal.pone.0143234 PMC4674107

[jkab282-B63] Schmieder R , Edwards R. 2011. Quality control and preprocessing of metagenomic datasets. Bioinformatics. 27:863–864. 21278185 10.1093/bioinformatics/btr026 PMC3051327

[jkab282-B64] Schornack S , Huitema E, Cano LM, Bozkurt TO, Oliva R, et al 2009. Ten things to know about oomycete effectors. Mol Plant Pathol. 10:795–803. 19849785 10.1111/j.1364-3703.2009.00593.x PMC6640533

[jkab282-B65] Schornack S , van Damme M, Bozkurt TO, Cano L, Smoker M, et al 2010. Ancient class of translocated oomycete effectors targets the host nucleus. Proc Natl Acad Sci USA. 107:17421–17426. 20847293 10.1073/pnas.1008491107 PMC2951462

[jkab282-B66] Seppey M , Manni M, Zdobnov EM. 2019. BUSCO: assessing genome assembly and annotation completeness. Methods Mol Biol.1962: 227-245. 10.1007/978-1-4939-9173-0_14 31020564

[jkab282-B67] Stamler RA , Sanogo S, Goldberg NP, Randall JJ. 2016. Phytophthora species in rivers and streams of the southwestern United States. Appl Environ Microbiol. 82:4696–4704. 27235435 10.1128/AEM.01162-16 PMC4984303

[jkab282-B68] Studholme DJ , McDougal RL, Sambles C, Hansen E, Hardy G, et al 2016. Genome sequences of six Phytophthora species associated with forests in New Zealand. Genom Data. 7:54–56. 26981359 10.1016/j.gdata.2015.11.015 PMC4778589

[jkab282-B69] Studholme DJ , Panda P, Sanfuentes Von Stowasser E, González M, Hill R, et al 2019. Genome sequencing of oomycete isolates from Chile supports the New Zealand origin of *Phytophthora kernoviae* and makes available the first Nothophytophthora sp. genome. Mol Plant Pathol. 20:423–431. 30390404 10.1111/mpp.12765 PMC6637878

[jkab282-B70] Thorpe P , Cock PJ, Bos JIB. 2016. Comparative transcriptomics and proteomics of three different aphid species identifies core and diverse effector sets. BMC Genomics. 17:172. 26935069 10.1186/s12864-016-2496-6 PMC4776380

[jkab282-B71] Thorpe P , Escudero-Martinez CM, Cock PJA, Eves-van den Akker S, Bos JIB. 2018. Shared transcriptional control and disparate gain and loss of aphid parasitism genes. Genome Biol Evol. 10:2716–2733. 30165560 10.1093/gbe/evy183 PMC6186164

[jkab282-B72] Tyler BM. 2007. *Phytophthora sojae*: root rot pathogen of soybean and model oomycete. Mol Plant Pathol. 8:1–8. 20507474 10.1111/j.1364-3703.2006.00373.x

[jkab282-B73] Van Poucke K , Haegeman A, Goedefroit T, Focquet F, Leus L, et al 2021. Unravelling hybridization in Phytophthora using phylogenomics and genome size estimation. IMA Fungus. 12:16. 34193315 10.1186/s43008-021-00068-w PMC8246709

[jkab282-B74] Vetukuri RR , Tripathy S, Malar CM, Panda A, Kushwaha SK, et al 2018. Draft genome sequence for the tree pathogen *Phytophthora plurivora*. Genome Biol Evol. 10:2432–2442. 30060094 10.1093/gbe/evy162 PMC6152947

[jkab282-B75] Wang S , Boevink PC, Welsh L, Zhang R, Whisson SC, et al 2017. Delivery of cytoplasmic and apoplastic effectors from *Phytophthora infestans* haustoria by distinct secretion pathways. New Phytol. 216:205–215. 28758684 10.1111/nph.14696 PMC5601276

[jkab282-B76] Wang T , Gao C, Cheng Y, Li Z, Chen J, et al 2020. Molecular diagnostics and detection of Oomycetes on fiber crops. Plants (Basel). 9:E769. 32575466 10.3390/plants9060769 PMC7355704

[jkab282-B77] Wawra S , Trusch F, Matena A, Apostolakis K, Linne U, et al 2017. The RxLR motif of the host targeting effector AVR3a of *Phytophthora infestans* is cleaved before secretion. Plant Cell. 29:1184–1195. 28522546 10.1105/tpc.16.00552 PMC5502441

[jkab282-B78] Weiß CL , Pais M, Cano LM, Kamoun S, Burbano HA. 2018. nQuire: a statistical framework for ploidy estimation using next generation sequencing. BMC Bioinformatics. 19:122. 29618319 10.1186/s12859-018-2128-z PMC5885312

[jkab282-B79] Whisson SC , Boevink PC, Moleleki L, Avrova AO, Morales JG, et al 2007. A translocation signal for delivery of oomycete effector proteins into host plant cells. Nature. 450:115–118. 17914356 10.1038/nature06203

[jkab282-B80] Win J , Morgan W, Bos J, Krasileva KV, Cano LM, et al 2007. Adaptive evolution has targeted the C-terminal domain of the RXLR effectors of plant pathogenic oomycetes. Plant Cell. 19:2349–2369. 17675403 10.1105/tpc.107.051037 PMC2002621

[jkab282-B81] Yang X , Tyler BM, Hong C. 2017. An expanded phylogeny for the genus Phytophthora. IMA Fungus. 8:355–384. 29242780 10.5598/imafungus.2017.08.02.09 PMC5729717

[jkab282-B82] Zhang H , Yohe T, Huang L, Entwistle S, Wu P, et al 2018. dbCAN2: a meta server for automated carbohydrate-active enzyme annotation. Nucleic Acids Res. 46:W95–W101. 29771380 10.1093/nar/gky418 PMC6031026

