# Supplementary material for: Draft genome assemblies for tree pathogens Phytophthora pseudosyringae and Phytophthora boehmeriae
Source: G3 (Bethesda). 2021 Aug 13;11(11):jkab282. doi: 10.1093/g3journal/jkab282 (PMC8527500; doi:10.1093/g3journal/jkab282)
Supplement: jkab282_Supplementary_Data [file jkab282_supplementary_data.zip › GENETICS-G3-2021-402693-s01.docx]

**Figure S1.** Percentage identity (green), bit score (blue) and alignment length (red) of *P. boehmeriae* (top) and *P. pseudosyringae* (bottom) proteins when BLASTP searched against GenBank non-redundant sequences. Percentage identity, bit score or alignment length (x axis) are plotted against number in each bin (y axis) for RXLR effector proteins, and entire predicted proteome. Note that the scales for bit score and alignment length differ between the plots of RXLR effector proteins and total proteome.
