# Supplementary figures and images for: Draft genome assemblies for tree pathogens Phytophthora pseudosyringae and Phytophthora boehmeriae
Source: G3 (Bethesda). 2021 Aug 13;11(11):jkab282. doi: 10.1093/g3journal/jkab282 (PMC8527500; doi:10.1093/g3journal/jkab282)

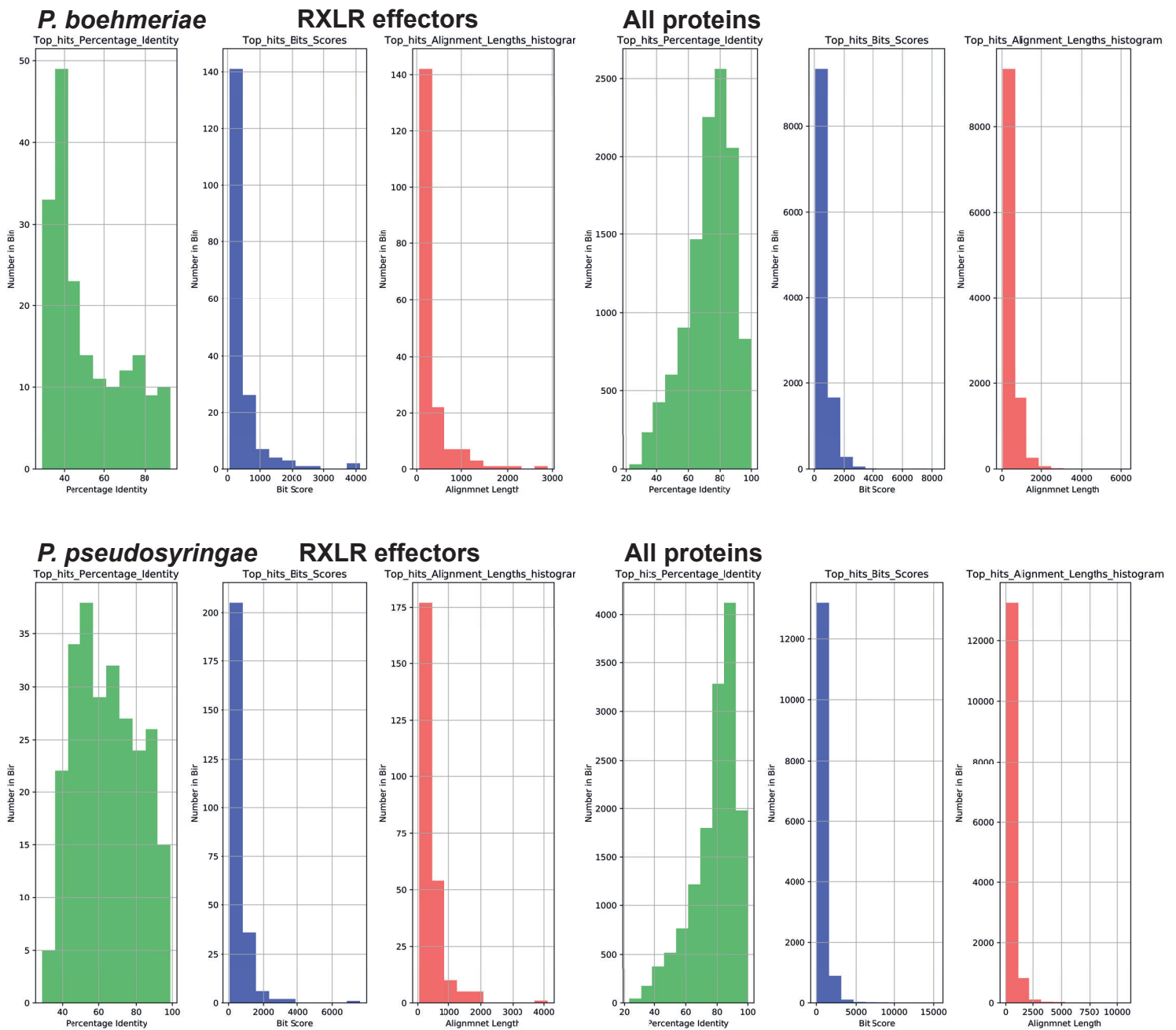

Thorpe et al. Supplementary Figure 1

Supplement: jkab282_Supplementary_Data [file jkab282_supplementary_data.zip › GENETICS-G3-2021-402693-s02.pdf]
